# Evaluating the Safety of Retrograde Intrarenal Surgery (RIRS): Intra- and Early Postoperative Complications in Patients Enrolled in the Global Multicentre Flexible Ureteroscopy Outcome Registry (FLEXOR)

**DOI:** 10.1590/S1677-5538.IBJU.2024.0055

**Published:** 2024-05-20

**Authors:** Carlo Giulioni, Demetra Fuligni, Carlo Brocca, Deepak Ragoori, Ben Hall Chew, Esteban Emiliani, Chin Tiong Heng, Yiloren Tanidir, Nariman Gadzhiev, Abhishek Singh, Saeed Bin Hamri, Boyke Soehabali, Andrea Benedetto Galosi, Thomas Tailly, Olivier Traxer, Bhaskar Kumar Somani, Marcelo L. Wroclawski, Vineet Gauhar, Daniele Castellani

**Affiliations:** 1 Urology Unit Azienda Ospedaliero-Universitaria delle Marche Università Politecnica delle Marche Ancona Italy Urology Unit, Azienda Ospedaliero-Universitaria delle Marche, Università Politecnica delle Marche, Ancona, Italy;; 2 Department of Urology Asian Institute of Nephrology & Urology Hyderabad Telangana India Department of Urology, Asian Institute of Nephrology & Urology, Irram Manzil Colony, Hyderabad, Telangana, India;; 3 Department of Urology University of British Columbia Vancouver Canada Department of Urology, University of British Columbia, Vancouver, Canada;; 4 Department of Urology Fundacion Puigvert Autónomos University of Barcelona Barcelona Spain Department of Urology, Fundacion Puigvert, Autónomos University of Barcelona, Barcelona, Spain;; 5 Department of Urology Ng Teng Fong General Hospital Singapore Singapore Department of Urology, Ng Teng Fong General Hospital, Singapore, Singapore;; 6 Department of Urology School of Medicine Marmara University Istanbul Turkey Department of Urology, School of Medicine, Marmara University, Istanbul, Turkey;; 7 Department of Urology Saint-Petersburg State University Hospital Saint-Petersburg Russia Department of Urology, Saint-Petersburg State University Hospital, Saint-Petersburg, Russia;; 8 Muljibhai Patel Urological Hospital Nadiad India Muljibhai Patel Urological Hospital, Nadiad, India;; 9 Department of Surgery Ministry of the National Guard Health Affairs King Saud Bin Abdulaziz University for Health Sciences Riyadh Saudi Arabia Division of Urology, Department of Surgery, Ministry of the National Guard Health Affairs, King Saud Bin Abdulaziz University for Health Sciences, King Abdullah International Medical Research Center, Riyadh, Saudi Arabia;; 10 Abdul Wahab Sjahranie Hospital Medical Faculty Mulawarman University Samarinda Indonesia Department of Urology, Abdul Wahab Sjahranie Hospital, Medical Faculty Mulawarman University, Samarinda, Indonesia;; 11 Department of Urology University Hospital Ghent Ghent Belgium Department of Urology, University Hospital Ghent, Ghent, Belgium;; 12 Department of Urology AP-HP Sorbonne University Tenon Hospital Paris France Department of Urology AP-HP, Sorbonne University, Tenon Hospital, Paris, France;; 13 Department of Urology University Hospitals Southampton Southampton UK Department of Urology, University Hospitals Southampton, NHS Trust, Southampton, UK;; 14 Department of Urology Hospital Israelita Albert Einstein São Paulo SP Brasil Department of Urology, Hospital Israelita Albert Einstein, São Paulo, SP. Brasil

**Keywords:** Kidney Calculi, Hematuria, Sepsis

## Abstract

**Purpose:**

To assess the incidence of the most common intra- and early postoperative complications following RIRS in a large series of patients with kidney stones.

**Methods:**

We conducted a retrospective analysis of patients with kidney stones who underwent RIRS across 21 centers from January 2018 to August 2021, as part of the Global Multicenter Flexible Ureteroscopy Outcome (FLEXOR) Registry.

**Results:**

Among 6669 patients undergoing RIRS, 4.5% experienced intraoperative pelvicalyceal system bleeding without necessitating blood transfusion. Only 0.1% of patients, required a blood transfusion. The second most frequent intraoperative complication was ureteric injury due to the ureteral access sheath requiring stenting (1.8% of patients). Postoperatively, the most prevalent early complications were fever/infections requiring antibiotics (6.3%), blood transfusions (5.5%), and sepsis necessitating intensive care unit admission (1.3%). In cases of ureteric injury, a notably higher percentage of patients exhibited multiple stones and stone(s) in the lower pole, and these cases were correlated with prolonged lasing and overall surgical time. Hematuria requiring a blood transfusion was associated with an increased prevalence of larger median maximum stone diameters, particularly among patients with stones exceeding 20 mm. Furthermore, these cases exhibited a significant prolongation in surgical time. Sepsis necessitating admission to the intensive care unit was more prevalent among the elderly, concomitant with a significantly larger median maximum stone diameter.

**Conclusions:**

Our analysis showed that RIRS has a good safety profile but bleeding requiring transfusions, ureteric injury, fever, and sepsis are still the most common complications despite advancements in technology.

## INTRODUCTION

The global incidence of kidney stone disease (KSD) is increasing worldwide, with an estimated lifetime prevalence of 14% ( 1 ), leading to a simultaneous rise in annual curative procedures ( 2 ). The most common procedures employed to manage stones in the upper tract are shockwave lithotripsy, percutaneous nephrolithotomy, and retrograde intrarenal surgery (RIRS) ( 3 ).

The ongoing advancements in endoluminal endourology for the upper urinary tract have broadened the scope of RIRS in clinical practice. RIRS has moved from an alternative to primary intervention for treating renal stones up to 20 mm in maximum diameter, according to the European and American Association of Urology guidelines ( 3 , 4 ). However, akin to any surgery, even this one exposes the patient to a series of complications. The most commonly reported complications include discomfort from a ureteral stent, injuries to the ureteral wall, and migration of fragments ( 5 ).

Among early worst postoperative complications, the most prevalent is fever, followed by urinary tract infections, with an incidence ranging from 0.2% to 15% ( 5 ). Another particularly serious complication is sepsis, which has an incidence between 0.1 and 4.3% ( 6 ). In rare cases, sepsis can become fatal and represents the primary cause of postoperative mortality, alongside other complications such as cardiac issues, respiratory complications, multiorgan failure, and hemorrhagic complications ( 5 ).

This study aims to evaluate both intra and early postoperative complications in a large, multicenter series of patients undergoing RIRS for kidney stones.

## MATERIALS AND METHODS

A retrospective analysis of adult patients undergoing RIRS for kidney stones from January 2018 to August 2021 in 21 centers from fifteen countries was performed. The full study protocol has been previously published ( 7 ). Briefly, the FLEXible ureteroscopy Outcomes Registry (FLEXOR) study enrolled 6669 patients who underwent RIRS for unilateral kidney stone(s). Exclusion criteria were bilateral procedures, ureteral stone, renal anomalies, RIRS done prone position, or RIRS done as combined procedures for Endoscopic Combined Intrarenal Surgery. Participating surgeons had previous experience performing at least 500 flexible ureteroscopy cases. There were no specific criteria established for including or excluding particular methods of performing the procedure. The RIRS procedure was conducted based on the standard of care and surgical practices followed by each institute. Antibiotic prophylaxis was performed according to each center’s protocol. Intraoperative outcomes and stone-free rate were already reported ( 7 ) and are not analyzed in this paper. For the present study, both intraoperative and early postoperative complications were analyzed. Early postoperative complications were considered if occurred within 30 days of RIRS and classified according to the Clavien-Dindo classification system. Fever was defined as an increase in body temperature ≥38°C. Sepsis was defined as “a life-threatening organ dysfunction caused by a dysregulated host response to infection” and assessed using the quick Sequential Organ Failure Assessment (q-SOFA) score ( 8 ).

The participant centers contributed to the database established, maintained, and overseen in accordance with the approval obtained from the Institutional board review of the leading centers (#AINU 08/2022). Each participating center had its own approval.

### Statistical Analysis

Patients were divided into two groups according to the presence or not of each complication, namely ureteric injury, sepsis, and hematuria requiring a blood transfusion. Continuous data were reported as mean and standard deviation and categorical data were displayed as absolute numbers and percentages. In cases where data were absent, the count of patients with missing values was indicated. Hence, the percentages were computed considering the entire cohort. The Student t-test was employed to examine differences between the two groups concerning continuous variables, while Chi-square and Fisher’s exact test were utilized for categorical variables. Statistical tests were conducted using the SPSS software package version 25.0 (IBM Corp., Armonk, NY).

## RESULTS

In Table-1 shows intra and early postoperative complications. 300 (4.5%) patients had intraoperative pelvicalyceal system bleeding not requiring blood transfusion. The second most common intraoperative complication was ureteric injury due to ureteral access sheath (UAS) requiring prolonged stenting that occurred in 119 (1.8%) patients. Intraoperative pelvicalyceal bleeding requiring a blood transfusion occurred in 6 (0.1%) patients. Fever/Infections requiring antibiotics (Clavien grade 2) was the most common postoperative complication that happened in 407 (6.3%) patients. 366 (5.5%) patients required a blood transfusion (Clavien grade 2), while sepsis with intensive care unit admission (Clavien grade 4b) occurred in 84 (1.3%) patients. Other complications occurred in 12 (0.18%) patients.

**Table 1:** Overall intra- and early postoperative complications.

Complication	N=6669
**Intraoperative**	
Pelvicalyceal system bleeding not requiring blood transfusion	300 (4.5%)
Pelvicalyceal system bleeding requiring blood transfusion	6 (0.1%)
Ureteric injury due to access sheath requiring stenting	119 (1.8%)
**Early Postoperative**	
Fever/Infections requiring antibiotics (Clavien grade 2)	407 (6.3%)
Hematuria requiring blood transfusions (Clavien Grade 2)	366 (5.5%)
Sepsis requiring ICU admission (Clavien Grade 4)	84 (1.3%)
**Others**	**12 (0.18%)**
Aspiration pneumonia with acute respiratory failure (Clavien grade 3)	1 (0.01%)
Urinary retention requiring bladder catheterization (Clavien grade 1)	4 (0.06%)
Perirenal Urinoma requiring drainage (Clavien grade 3b)	1 (0.01%)
Perirenal hematoma requiring drainage (Clavien grade 3b)	1 (0.01%)
Stent migration requiring reinsertion (Clavien grade 3b)	5 (0.07%)

Table-2 shows data on patients having or not having ureteric injury. Median age and maximum stone diameter, male gender, prestented patients, use of semirigid ureteroscopy before RIRS, use of reusable scopes (vs single-use scopes), Holmium laser (vs Thulium fiber laser) and general anesthesia (vs spinal anesthesia) were similar between the groups. Conversely, there was a significantly larger proportion of patients with multiple stones (58.8% vs 41.5%, p<0.001) and stones in the lower pole (63.5% vs 44.1%) in patients with ureteric injury. Patients with injury had a significantly longer median lasing [27 (24-31.5) vs 22 ( 14 -35) minutes, p=0.020) and total operation times [65 (49.5-90) vs 54 (39-75) minutes, p<0.001].

**Table 2:** Patients’ characteristics according to the occurrence or not of ureteric injury requiring prolonged stenting.

	Ureteric injury N=119 (1.8%)	No ureteric injury N=6550 (98.2%)	p
Age, median [IQR]	48 [38, 58.5]	49 [37, 60]	0.510
Proportion of Females	41 (34.5)	2221 (33.9)	0.979
Presenting with fever	26 (21.8)	625 (9.6)	<0.001
Urine culture positive	30 (25.2)	2358 (36.6)	0.013
Multiple stones	67 (58.8)	2667 (41.5)	<0.001
Maximum stone diameter (mm), median [IQR]	11 [8.0, 14]	11.0 [8.0, 15]	0.376
Stone diameter >2cm	2 (4.9)	298 (10.3)	0.382
Stone location*			
Upper pole	24 (20.9)	1450 (22.3)	0.797
Interpolar	29 (25.2)	2015 (31.0)	0.222
Lower pole	73 (63.5)	2873 (44.1)	<0.001
Pelvis	40 (33.9)	2156 (33.1)	0.926
HU, median [IQR]	845 [610, 1168]	1000 [750, 1200]	0.006
Prestented patients	65 (54.6)	3093 (47.3)	0.133
Preoperative antibiotics	102 (85.7)	5027 (76.7)	0.028
Reusable scope	87 (73.1)	4720 (72.1)	0.881
Holmium laser	89 (74.8)	4735 (72.3)	0.617
Total operation time, median [IQR]	65 [49.5, 90]	54 [39, 75]	<0.001
Lasing time, median [IQR]	27 [24, 31.5]	22 [14, 35]	0.020
General anesthesia	83 (69.7)	4270 (65.2)	0.348
**Respiratory control**			**0.302**
None	65 (54.6)	3223 (49.2)	
Gated	39 (32.8)	2171 (33.1)	
Apneic	15 (12.6)	1156 (17.6)	

HU = Hounsfield unit; IQR = interquartile range; * = more than one choice possible

Table-3 shows data on patients having or not having hematuria requiring a blood transfusion. Median age and proportion of males did not differ significantly. Median maximum stone diameter [15 ( 12 - 20 ) vs 11 (8.0-15) mm, p<0.001] was significantly higher in patients requiring a blood transfusion. There was a significantly higher proportion of patients having a stone larger than 20 mm (37.2% vs 8.9%, p<0.001) in patients who had blood transfusions. There was a significantly higher proportion of pre-stented patients in the non-transfusion group (47.9% vs 38.6%, p=0.001). The use of a reusable scope was significantly higher in the non-transfusion group (73.0% vs 53.8%, p<0.001). Surgical time was also significantly longer in patients having blood transfusion [65 (48-85) vs 52 (39-75) minutes, p<0.001].

**Table 3:** Patients characteristics according to the occurrence or not of hematuria requiring a blood transfusion.

	Hematuria N=366 (5.5%)	No hematuria N=6303 (94.5%)	p
Age, median [IQR]	47 [38, 58]	49 [37, 60]	0.241
Proportion of Females	129 (35.2)	2133 (33.8)	0.621
Presenting with fever	51 (13.9)	600 (9.6)	0.008
Urine culture positive	96 (26.3)	2292 (37.0)	<0.001
Multiple stones	179 (50.0)	2555 (41.3)	0.001
Maximum stone diameter (mm), median [IQR]	15 [12, 20]	11 [8.0, 15]	<0.001
Stone diameter >2cm	51 (37.2)	249 (8.9)	<0.001
Stone location*			
Upper pole	105 (29.3)	1369 (21.9)	0.001
Interpolar	116 (32.4)	1928 (30.8)	0.555
Lower pole	181 (50.4)	2765 (44.1)	0.023
Pelvis	153 (42.4)	2043 (32.5)	<0.001
HU, median [IQR]	900 [588, 1100]	1000 [750, 1208]	<0.001
Prestented patients	141 (38.6)	3017 (47.9)	0.001
Preoperative antibiotics	284 (77.6)	4845 (76.9)	0.797
Reusable scope	208 (56.8)	4599 (73.0)	<0.001
Holmium laser	249 (68.0)	4575 (72.6)	0.067
Total operation time, median [IQR]	65 [48, 85]	52 [39, 75]	<0.001
Lasing time, median [IQR]	20 [12, 30]	22 [15, 35]	0.244
General anesthesia	241 (65.8)	4112 (65.2)	0.856
**Respiratory control**			**0.023**
None	199 (54.4)	3089 (49.0)	
Gated	121 (33.1)	2089 (33.1)	
Apneic	46 (12.6)	1125 (17.8)	

HU = Hounsfield unit; IQR = interquartile range; * = more than one choice possible

Table-4 shows data on patients having or not having sepsis requiring intensive care unit admission. Patients who had sepsis were significantly older [54 (40-66) vs 49 (37-60) years, p=0.024], whilst the proportion of females did not significantly differ. There was a significantly higher proportion of patients presenting with fever in the sepsis group (21.7% vs 9.6%, p=0.001). Median maximum stone diameter [15 ( 10 - 20 ) vs 11 (8.0-15 mm, p<0.001] was significantly higher in the sepsis group. Yet, there was a significantly higher proportion of patients having a stone larger than 20 mm in patients who had sepsis (24.5% vs 9.9%, p<0.001). The use of a reusable scope was significantly higher in the non-sepsis group (60.7% vs 72.2%, p=0.027). Lasing and total surgical time did not differ significantly.

**Table 4:** Patients characteristics according to the occurrence or not of sepsis requiring intensive care admission.

	Sepsis N=84 (1.3%)	No sepsis N=6585 (98.7%)	p
Age, median [IQR]	54 [40, 66]	49 [37, 60]	0.024
Proportion of Females	33 (39.3)	2229 (33.8)	0.352
Presenting with fever	18 (21.7)	633 (9.6)	0.001
Urine culture positive	30 (35.7)	2358 (36.5)	0.980
Multiple stones	32 (38.1)	2702 (41.8)	0.567
Maximum stone diameter (mm), median [IQR]	15 [10, 20]	11 [8.0, 15]	<0.001
Stone diameter >2cm	13 (24.5)	287 (9.9)	0.001
Stone location*			
Upper pole	20 (23.8)	1454 (22.3)	0.839
Interpolar	30 (35.7)	2014 (30.8)	0.395
Lower pole	33 (39.3)	2913 (44.5)	0.393
Pelvis	26 (31.0)	2170 (33.1)	0.764
HU, median [IQR]	1027 [776, 1220]	1000 [746, 1200]	0.603
Prestented patients	38 (45.2)	3120 (47.4)	0.774
Preoperative antibiotics	62 (73.8)	5067 (76.9)	0.584
Reusable scope	51 (60.7)	4756 (72.2)	0.027
Holmium laser	46 (54.8)	4778 (72.6)	<0.001
Total operation time, median [IQR]	60 [33, 87.5]	55 [40, 76]	0.364
Lasing time, median [IQR]	28 [15.5, 45.5]	22 [14, 35]	0.129
General anesthesia	38 (45.2)	4315 (65.5)	<0.001
**Respiratory control**			0.119
None	48 (57.1)	3240 (49.2)	
Gated	19 (22.6)	2191 (33.3)	
Apneic	17 (20.2)	1154 (17.5)	

HU = Hounsfield unit; IQR = interquartile range; * = more than one choice possible

## DISCUSSION

RIRS gained prominence in the management of renal stones and is currently recommended for kidney stones measuring up to 2 cm in the largest diameter ( 9 ). In current practice, RIRS is regarded as an outpatient procedure due to its safety ( 10 , 11 ). The advancement of technology and instruments employed has also heightened the effectiveness of this surgical procedure. However, in the endeavor to expand the RIRS indications based on both patient and stone characteristics, it is crucial to carefully assess the risks associated with this procedure. Indeed, maintaining adequate intrarenal pressure and temperature is imperative to prevent epithelial damage of the collecting system and intrarenal bacterial migration by retrorenal reflux ( 12 ). Furthermore, while the percentages remain low, there is an increasing trend in post-RIRS deaths over the past decade, with the etiology of sepsis identified in over half of the cases ( 13 ).

In our analysis, encompassing 6669 patients, we examined the most prevalent intra- and postoperative complications. Ureteric injury due to UAS occurred in 1.8% of patients. This ancillary device is widely used in the urologist’s armamentarium for RIRS owing to its procedural benefits. Indeed, its utilization enhances irrigation efflux during RIRS and mitigates intrarenal pressure in the context of forced irrigation ( 14 ). As evidenced by an in-vitro investigation, in the absence of a UAS, the intrarenal pressure (IRP) surpassed 40 cmH2O at irrigation levels of 153 cmH2O and 193 cmH2O, reaching up to 165 cmH2O ( 15 ) using an automated irrigation system. In contrast, the employment of a UAS consistently kept IRP below 40 cmH2O across diverse irrigation pressures. Notwithstanding, the introduction of a UAS entails the prospect of acute ureteral injury, attributed to its caliber being 4 to 8 French larger than the typical adult ureter, thereby resulting in an undue application of bulking force ( 16 ). Moreover, research indicates that subjecting a UAS to an elevated deployment force heightens the likelihood of ureteric injury ( 17 ). Consequently, surgeons should exercise caution to prevent excessive insertion force during placement. It is plausible that pre-stenting may confer additional advantages in this context.

Interestingly, when comparing patients who experienced ureteral injury with those who did not, it was observed in our series that the former group exhibited a higher prevalence of preoperative antibiotic usage to treat positive urine culture. This finding suggests alterations in the ureteral mucosa as a consequence of chronic urinary tract infection. Specifically, upon scrutinizing specimens of the ureter colonized by Escherichia coli, an upregulation in the expression of genes of epithelial cells associated with the promotion of the apoptosis process is observed ( 18 ).

The multiplicity and location of stones in the lower pole were found to be more prevalent, and the overall operation and laser times were on average higher in patients with ureteral injuries. The characteristics of the stone, encompassing its location, size, and number, delineate the surgical complexity, exerting an influence on treatment duration and being correlated with the development of complications, such as inability to reach the stone, hematuria, ureteral lesions, infectious complications and retreatment ( 19 ). Our findings are consistent with a study that included 1060 patients undergoing routine upper tract imaging eight weeks after ureteroscopy ( 20 ). Factors identified as predictors for postoperative hydronephrosis included a larger stone size and extended operative time.

In the FLEXOR database, sepsis requiring intensive care unit admission occurred in 84 patients (1.3%). The mean age of patients with this complication was higher compared to those without it, although controversies have emerged on this topic. A sub-analysis of elderly patients from the FLEXOR database did not reveal age as a predictive factor for overall complications or the risk of postoperative sepsis ( 21 ). Yet, a recent systematic review also indicated that most studies found insignificant relationships between age and infectious complications following RIRS ( 22 ). Therefore, one could argue that chronological age itself does not increase the risk of sepsis when considering the higher likelihood of having comorbidities. Indeed, it has been demonstrated that the presence of chronic pathological conditions has a significant negative impact on surgical risk ( 23 ). Furthermore, these patients might require more frequently the use of general anesthesia, which would explain the higher frequency of the latter among patients with postoperative septic complications in our analysis. The stone maximum diameter was found to be higher in patients with sepsis, with stones larger than 2 cm being more frequent in those who had sepsis in our cohort. Indeed, larger stone sizes entail prolonged operative times and increased laser utilization, leading to elevated intrarenal pressure and temperature. This can result in injury to the mucosa of the collecting system and the reabsorption of irrigation fluid containing bacteria and endotoxins ( 24 ). Our findings align with the review by Corrales et al., which particularly emphasized stone size as a potential risk factor for sepsis, asserting that the risk of sepsis increases with stone size ( 6 ). In our series, patients with sepsis requiring ICU admission exhibited a lower incidence of Holmium laser and reusable scope usage but there is no conclusive evidence suggesting that they are independent predictive factors for infective complications. However, essential details such as the lasing settings, laser power, lithotripsy type (dusting vs. fragmentation), and any encountered issues during the lithotripsy procedure are unavailable, preventing the execution of any subset analysis. Notably, literature does not address these two variables in the context of the risk of post-RIRS sepsis ( 6 , 22 ). Therefore, additional research is warranted to validate these findings.

FLEXOR registry documented a 4.5% incidence of mild collecting system injuries accompanied by hematuria that did not require blood transfusion, whereas only 0.1% of cases required transfusion, a phenomenon likely influenced by various factors such as stone complexity, collecting system intricacy, laser parameters, and the surgeon’s intraoperative proficiency. In contrast, our study revealed that 5.5% of patients received a blood transfusion for unspecified reasons. Regrettably, detailed information regarding the etiology of these transfusions was unavailable. The dataset lacked information on patients with a history of anticoagulant or antiplatelet usage, underlying coagulopathy, or chronic anemia. Additionally, there was a dearth of specifics regarding whether blood transfusions were directly linked to complications arising from RIRS or were administered as part of managing other comorbidities. Unfortunately, the data did not include information on procedural complexities that might have necessitated transfusions. While the study reported one case each of perirenal hematoma and urinoma ( Table-1 ), details regarding whether these complications required transfusion and their subsequent outcomes were not available. Notably, these complications are recognized as reported outcomes of RIRS, often attributed to elevated IRP or potential surgeon negligence in laser use or overlooking collecting system injuries during RIRS, which may manifest either immediately postoperatively or later ( 25 , 26).

Hematuria requiring a blood transfusion occurred in 5.5% of patients in our series. The restricted diameter of the RIRS scope compounds the intricacies of the surgical procedure, primarily attributable to the onset of hematuria. Factors contributing to the heightened incidence of bleeding during these interventions include increased IRP, distension of cavities in the renal pelvis and calyces, urothelial injury from direct laser application, and stone fragmentation. Our comparative analysis revealed that stone characteristics, specifically their multiplicity and maximum diameter, along with the operative time, played a role in the hematuria rate. Kim et al. demonstrated a correlation between the severity of hematuria and stone size in univariate analysis and operative times in multivariate analysis within complex stone classifications ( 27 ).

The relationship between reusable scopes and lower rates of hemorrhagic complications remains uncertain. Nevertheless, the application of specific disposable scopes introduced heightened complexities in navigating the pelvicalyceal system, exacerbating renal mucosal trauma ( 28 ). Furthermore, the imperative for more extensive irrigation to enhance visual clarity consequently might lead to an increased incidence of hematuria.

This study is subject to certain limitations. Firstly, the retrospective design of the investigation imposes inherent constraints on establishing causal relationships and controlling for potential confounding variables. Secondly, the study’s focus on a 30-day complication period restricts the comprehensive exploration of long-term outcomes and the identification of potential evolving trends beyond the specified timeframe. Furthermore, as previously highlighted, the absence of comprehensive data hinders a thorough comprehension of the underlying reasons and processes leading to intra and post-operative occurrences of injury, sepsis, and bleeding. Such detailed data would have provided invaluable insights for enhancing the safety protocols of the procedure. Despite these data limitations, our registry analysis unequivocally demonstrates the safety and effectiveness of RIRS when performed by experienced practitioners. It is imperative, however, to acknowledge certain constraints and uphold defined boundaries to mitigate the risk of serious complications. Regrettably, comprehensive data on the site of injury, the classification of UAS injuries according to the prospective analysis by Traxer and Thomas ( 29 ), and any inadvertent ureteral injuries resulting from miscellaneous instrument usage were not reported. Additionally, long-term sequelae associated with UAS usage or follow-up information on patients with injuries beyond the 30-day mark are unavailable. It is noteworthy, however, that no instances of ureteric avulsions were documented in our series, and all reported ureteric injuries were managed solely with stent placement, with no recorded instances of surgical repairs but all patients should be followed long-term, because of the higher risk of ureteral stenosis. Lastly, the limited time frame precludes the assessment of the development of postoperative ureteral stenosis, another complication that is relatively commonly encountered.

## CONCLUSIONS

FLEXOR reaffirms the safety of RIRS as a minimally invasive surgery for stones. Our study showed that bleeding requiring transfusions, ureteric injury, fever, and sepsis are still the most common complications despite advancements in technology. Experience alone is not enough to prevent but can definitively mitigate serious adverse surgical complications and patients should be made aware of all possible complications.
